# Digital soil mapping in the Bara district of Nepal using kriging tool in ArcGIS

**DOI:** 10.1371/journal.pone.0206350

**Published:** 2018-10-26

**Authors:** Dinesh Panday, Bijesh Maharjan, Devraj Chalise, Ram Kumar Shrestha, Bikesh Twanabasu

**Affiliations:** 1 Department of Agronomy and Horticulture, University of Nebraska-Lincoln, Lincoln, Nebraska, United States of America; 2 Nepal Agricultural Research Council, Lalitpur, Nepal; 3 Institute of Agriculture and Animal Science, Lamjung Campus, Lamjung, Nepal; 4 Hexa International Pvt. Ltd., Lalitpur, Nepal; 5 Institute for Geoinformatics, Westfalische Wilhelms-Universitat Munster, Munster, Germany; US Department of Agriculture, UNITED STATES

## Abstract

Digital soil mapping has been widely used to develop statistical models of the relationships between environmental variables and soil attributes. This study aimed at determining and mapping the spatial distribution of the variability in soil chemical properties of the agricultural floodplain lands of the Bara district in Nepal. The study was carried out in 23 Village Development Committees with 12,516 ha total area, in the southern part of the Bara district. A total of 109 surface soil samples (0 to 15 cm depth) were collected and analyzed for pH, organic matter (OM), nitrogen (N), phosphorus (P, expressed as P_2_O_5_), potassium (K, expressed as K_2_O), zinc (Zn), and boron (B) status. Descriptive statistics showed that most of the measured soil chemical variables (other than pH and P_2_O_5_) were skewed and non-normally distributed and logarithmic transformation was then applied. A geostatistical tool, kriging, was used in ArcGIS to interpolate measured values for those variables and several digital map layers were developed based on each soil chemical property. Geostatistical interpolation identified a moderate spatial variability for pH, OM, N, P_2_O_5_, and a weak spatial variability for K_2_O, Zn, and B, depending upon the use of amendments, fertilizing methods, and tillage, along with the inherent characteristics of each variable. Exponential (pH, OM, N, and Zn), Spherical (K_2_O and B), and Gaussian (P_2_O_5_) models were fitted to the semivariograms of the soil variables. These maps allow farmers to assess existing farm soils, thus allowing them to make easier and more efficient management decisions and maintain the sustainability of productivity.

## Introduction

Applications of pedometric mapping, also called predictive mapping, i.e., the spatial prediction of soil variables at unobserved locations using statistical inference, have become increasingly important since their initial development in the early 1800s. The utility of such maps was due to the introduction of geostatistics, allowing researchers to accurately interpolate spatial patterns of soil properties [1]. One of the current versions of pedometric mapping, digital soil mapping (DSM), involves the creation and population of spatial soil information systems using field and laboratory observational methods coupled with spatial and non-spatial soil inference systems [2–5]. Field sampling is used to determine the spatial distribution of soil properties, and these surface grids point data are then interpolated to estimate soil properties in areas not sampled [6]. In contrast, existing conventional soil survey methods are relatively slow and expensive; therein, soil databases are neither exhaustive enough nor precise enough to promote an extensive and credible use of soil information within the spatial data [7].

Several statistical models can be used in DSM to develop a relationship between soil properties and environmental variables (often related to soil forming factors such as terrain attributes—altitude, aspect, and slope) rather than from soil observations alone [8], and McBratney *et al*. [9] thorough reviewed the various models used. Soil nutrients are one of the most important properties governing soil quality, and hence have a significant impact on the variability of soil productivity and crop production. The spatial variability of soil properties can be mapped using an interpolation technique [10]. Many spatial interpolation methods have been developed and several terms have been used to distinguish them, including “deterministic” and “stochastic” [11]. Deterministic interpolation methods such as thiessen, density estimation, inverse-distance-weighted, and splines, provide no assessment of errors, whereas stochastic interpolation and kriging methods do provide prediction error assessments.

Kriging is a geostatic interpolation technique that has proven sufficiently robust for estimating values at non-sampled locations based on sampled data. It provides the best linear unbiased estimates and information on the distribution of the estimation error and shows strong statistical advantages [12]. The use of the geostatistical interpolation technique also reduces the costs of field sampling and laboratory analysis, provided that a given set of soil samples sufficiently represents the study area [13]. However, the reliability of spatial variability maps depends on the adequate sampling data and the accuracy of the spatial interpolation [14].

There is a significantly increased trend in the use of DSM mainly due to recent advances in technology related to quantitative methodologies and geographic information systems. For example, spatial variability of organic matter (OM), pH, and potassium (K) were mapped using kriging by Lopez-Granados *et al*. [15] in a 40 ha field located in southern Spain. Santos Francés *et al*. used the kriging interpolation method for the production of spatial distribution of the heavy metal contents in the soils of northern Spain [16] and northern Peru [17], respectively. Balkovič *et al*. [18] reported that the DSM model represents a complete alternative to classical soil mapping at very fine scales on erosion affecting 37 ha of arable land in Slovakia, even when soil profile descriptions were collected merely by field estimation methods. In northwestern Australia, a DSM soil carbon map at the farm scale was developed from a total of 127 soil sampling locations in an area of 2300 ha [19]. Similarly, Zhang *et al*. [20] produced spatial variability maps of nitrogen (N), phosphorus (P) and K in winter wheat and summer maize in northeast China. Recently Zhu *et al*. [21] used an alternative DSM method, individual predictive soil mapping (iPSM), to map OM content in the topsoil layer of an area of 6000 ha in China and observed that iPSM is an effective alternative when existing soil samples are limited in their ability to fully represent the entire study area.

Despite the successful application of DSM in regions around the world, no single study has examined the use of DSM to represent soil nutrient variability in any part of Nepal. The commonly practiced soil fertility assessment is based on a random soil sampling protocol to obtain an average fertility value for a farmer’s field [22]. It ignores spatial variability, or those soil testing results that do not provide randomness of variations from one place to another. Consequently, some parts of the field may receive surplus fertilizer while others may lack nutrients and experience the undesired levels of productivity. The objective of this research was to determine and map the spatial distribution of variability in soil chemical properties of agricultural floodplain lands in the southern part of the Bara district of Nepal. The country’s economy relies heavily on agriculture and any breakthrough in soil mapping would immensely benefit farmers. Information on spatial variability of soil nutrients is also essential for sustainable management of soil fertility.

## Materials and methods

### Study area

**Fig 1:**
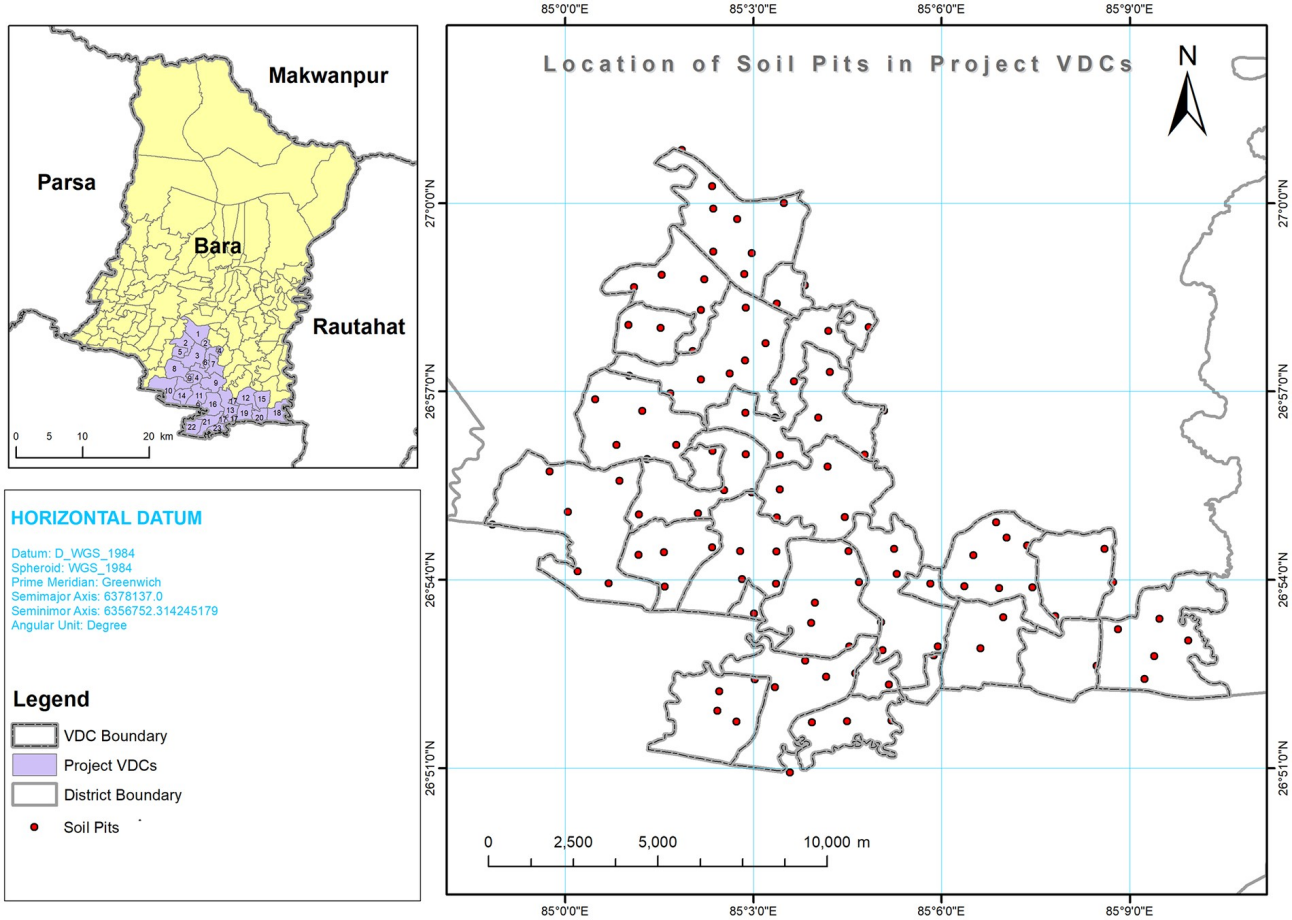
Study area in southern part of Bara district, Nepal which includes 23 Village Development Committees (VDCs). A large part of the study area lies in Terai region where the topographic variation ranges from 80 to 95 m, and climate is subtropical monsoon. A total of 109 soil samples were taken from the depth of 0–15 cm (topsoil layer) for determination of pH, OM, N, P_2_O_5_, K_2_O, Zn and B status on it.

Nepal is located in the south of Asia bordering neighbors of India and China and covers an area of approximately 147,181 km^2^. It has been divided broadly into three geographic regions: Himalayan, Hilly, and Terai. The study was conducted in the southern part of the Bara district, which falls under Terai region, and included the 23 Village Development Committees (VDCs) and covers 12,516 ha of land as shown in Fig 1. The topographic variation of the study area ranges from 80 to 95 m.

There are four seasons in Nepal: pre-monsoon (March to May), monsoon (June to September), post-monsoon (October to November) and winter (December to February). Monsoons are the Nepal’s main source of precipitation, accounting for 85% of the country’s total annual rainfall of 1800 mm, with the remaining 15% occurring in winter [23–24]. During a monsoon, all of the rivers are in spate, with bank-full discharges that cause flooding and inundation in several parts of the Terai region [25]. The study area becomes hottest (37 to 42°C) during the monsoon months compared to the country’s average temperature (28°C) due to seasonal changes and low altitude.

Rice (*Oryza sativa* L.) is the principal staple food of Nepal, accounting for about 67% of total cereal consumption. Most of the food crops for the entire country are grown in the Terai region, the granary of Nepal. The terai is generally made up of flat terrain with a hot, humid climate. About 80% of the land in this area is occupied by farmlands. Rice -wheat (*Triticum aestivum* L.)-fallow is the dominant cropping system in the study area followed by rice-wheat/lentils (*Lens culinaris*)-fallow, rice-wheat-maize (*Zea mays* L.), and sugarcane (*Sacharum officinarum*).

The soil association of the study area is developed by the changing river morphology. The soils have predominantly evolved from alluvial deposits and are dominated by sandy loam and silty clay, although clay loam and loamy sand are also present at considerable levels. It was observed that the majority of the area is occupied by land system unit 2b (deep alluvium: <0.5 degree slope, flat, imperfect drainage, sandy loam to silty clay, Aeric, Haplaquepts, Typic, and Fluventic) followed by 2a (deep alluvium: < 0.5 degree slope, depression, poor drainage, loam to silty clay, Aeric, Haplaquepts, and Typic), 3a (deep alluvium: < 1 degree slope, gently undulating, moderate drainage, sandy loam to silty clay, Haplaquepts, Typic, Ustocrepts, and Dystrochrepts), and 2c (stratified alluvium: < 1 degree slope, micro-relief, variable drainage, low areas subject to flooding, sandy loam to silty clay, Typic, and Fluventic).

### Soil sampling and analysis

Surface soil samples (0 to 15 cm depth) were collected during May 2013 using a soil auger in the study area. Soil sampling locations were selected to best represent the land use condition in each VDC while considering terrain attributes and drainage conditions. A few VDCs such as Parsurampur and Golganj had only one soil sampling location. By following one soil sample per location, a total of 109 soil samples were collected from the study area, and the details of soil sampling locations are given in Fig 1. A global positioning system receiver with 1 m precision was used to record the longitude and latitude of soil sampling locations. No specific permissions were required for soil sampling in these locations and the field studies did not involve endangered or protected species.

The collected soil samples were air-dried and sieved through a 2 mm sieve for chemical analysis conducted at the Regional Soil Testing Laboratory, Kaski district of Nepal. The soil chemical parameters tested and methods used are given in Table 1. Sodium bicarbonate (NaHCO_3_) and ammonium acetate (C_2_H_7_NO_2_) were used as the extractants for laboratory analysis of available phosphorus and potassium, respectively.

**Table 1:** Methods used for testing of soil chemical parameters at Regional Soil Testing Laboratory, Kaski district of Nepal.

Test	Method
pH	1:2 soil water suspension [26]
Organic matter content (OM, %)	Walkely and Black [27]
Total nitrogen content (N, %)	Kjeldahl [28]
Available phosphorus (P_2_O_5_, kg ha^-1^)	Olsen et al. [29]
Available potassium (K_2_O, kg ha^-1^)	Flame photometry [30]
Boron (B, mg kg^-1^)	Hot water method [31]
Zinc (Zn, mg kg^-1^)	DTPA [32]

### Statistical and geostatistical analysis

Descriptive statistics of soil properties, including mean, standard deviation, coefficient of variation, minimum, maximum, skewness (skew), and kurtosis, were calculated. For all measured soil characteristics, the visual method (histogram, boxplot and normal plot) and values of skew and kurtosis were used and no figures were included for the univariate test of normality in SAS software [33] prior to ordinary kriging.

This study focused on ordinary kriging (the general name for kriging), a linear geostatistical interpolation technique. Kriging estimates were calculated as weighted sums of the adjacent sampled concentrations. It is an improvement over inverse distance weighting (another geostatistical tool) interpolation because prediction estimates in kriging tend to be less biased and are accompanied by prediction standard errors [34]. Details of the kriging formula and calculation are given in Yao *et al*. [14]. The main application of geostatistics in soil science has been the estimation and mapping of soil attributes out of sampled areas [35].

Regardless of data distribution, kriging can provide the best-unbiased predictor of values at unsampled points, though data that have closer to a normal distribution can provide the best estimates of probability maps [36]. Therefore, it was necessary to normalize the dataset prior to geostatistical analysis because of high skew (Table 2) and the presence of outliers. Since the coefficient of skew was greater than one (except for pH and P_2_O_5_), the logarithmic transformation was applied for a kriging analysis (lognormal kriging, hereafter referred to as kriging) to stabilize the variance [35]. The logarithmic transformation resulted in smaller skew and kurtosis for OM, N, K_2_O, B, and Zn, and the transformed data passed the normality test.

**Table 2:** Summary statistical overview for selected soil chemical properties of study area (N = 109), including original and log transformed data (for skew and kurtosis).

Parameter	Mean	SD	CV	Min	Max	Skew (O)	Kurtosis (O)	Skew (T)	Kurtosis (T)
pH	6.4	1.02	16	4.2	8	-0.4	-0.9	-	-
OM (%)	2.13	1.5	70.33	0.15	5.98	1.16	0.44	-0.55	0.86
N (%)	0.11	0.07	70.56	0.01	0.3	1.2	0.51	-0.35	0.33
P_2_O_5_ (kg ha^-1^)	40.08	22	55.87	7	111	0.89	0.24	-	-
K_2_O (kg ha^-1^)	110.61	107	97.12	5	696	3.08	10.92	-0.36	3.2
Zn (mg kg^-1^)	0.08	0.06	77.42	0.01	0.42	2.1	7.16	-0.34	-0.14
B (mg kg^-1^)	1.03	0.44	42.35	0.67	5.15	4.73	21.23	-0.37	7.79

SD = standard deviation CV = coefficient of variation, Min = minimum, Max = maximum, skew = skewness. Skew (O) and Kurtosis (O) = skewness and kurtosis obtained from original data. Skew (T) and Kurtosis (T) = skewness and kurtosis obtained from log transformed data. Similar units for Mean, SD, Minimum, Maximum, Skew and Kurtosis, but % for CV.

A *dbf* file consisting of data for X and Y coordinates with respect to sampling site location was created in ArcGIS (version 10.2). Several digital map layers were then developed, using kriging in ArcMap, based on each soil chemical property at 1:25000 scale. The ranges for soil pH are classified as strongly acidic (<5.5), moderately acidic (5.5 to 6.2), neutral (6.2 to 7), moderately alkaline (7 to 7.8), and strongly alkaline (>7.8). Similarly, the rating charts for other soil parameters are given in Table 3, which is based on recommendations given by the Soil Management Directorate of the Department of Agriculture for the Terai region of Nepal [22].

**Table 3:** Range for different soil parameters given by the Soil Management Directorate, Department of Agriculture for Terai region of Nepal.

Range	OM (%)	N (%)	P_2_O_5_ (kg ha^-1^)	K_2_O (kg ha^-1^)	Zn (mg kg^-1^)	B (mg kg^-1^)
Very low	<1	<0.05	<10	<55	<0.25	<0.2
Low	1–2.5	0.05–0.1	10–30	55–110	0.25–0.5	0.2–0.5
Medium	2.5–5	0.1–0.2	30–55	110–280	0.5–1	0.5–1.2
High	5–10	0.2–0.4	55–110	280–500	1.0–2	1.2–2
Very high	>10	>0.4	>110	>500	>2	>2

Available P is expressed in P_2_O_5_ and available K in K_2_O, conversion factor: P_2_O_5_ = P*2.3 and K_2_O = K*1.2.

The kriging method uses semivariance to estimate the spatial distribution structure of the soil properties [37–38]. Semivariogram modeling and estimation are essential for structural analysis and spatial interpolation, which is akin to fitting a least-squares line in regression analysis [39]. It produces geostatistical parameters, including nugget, structural, sill, and range [38]. The spatial dependency (Sp. D) of soil parameters (the ratio of nugget to sill variances) is expressed as a percentage [40]. To ensure Sp. D, as a rule of thumb the sampling interval (lag) should be less than half of the range of the spatial variation [15]. If the ratio is less than 0.25, the variance has strong Sp. D and if the ratio ranges between 0.25 and 0.75, the variance has moderate Sp. D [41].

Moran’s I Index was used to measure spatial autocorrelation between sample points on the semivariagram cloud, which was evaluated using *z*-scores. Values greater than 1.96 or smaller than −1.96 are significant at *p* < 0.05 [42]. Similarly, the mean error (ME) and root mean square error (RMSE) was used for a cross-validation approach (or any given variogram model) to evaluate the accuracy or best fit of the kriging tool [43]. A ME value close to zero indicates that the interpolation method is unbiased. The lowest RMSE value indicates the best fit to the variogram model.

### Data analysis

Descriptive statistics and geostatistics were used to analyze the dataset, and descriptive statistics along with a normality test were run in SAS software. All maps were produced using GIS software ArcMap (version 10.2) and its spatial analyst and geostatistical analysis extensions. The structure of spatial variability was analyzed through semivariogram. Spatial distribution was analyzed through kriging interpolation.

## Results and discussion

### Descriptive statistics for soil chemical properties

The commonly used descriptive statistical summary of the pH, OM, N, P_2_O_5_, K_2_O, Zn, and B is presented in Table 2. The variability was interpreted using the coefficient of variation (CV) and classified into most (CV: >35%), moderate (CV: 15 to 35%) and least (CV: <15%) variable ranges [44]. The CV ranged from 16.0% (in pH) to 97.12% (in K_2_O). The range of CV for the soil sampling locations suggested different degrees of heterogeneity among the properties studied.

The pH values were ranging from 4.2 to 8 with a mean of 6.4, which was also similar to the median value of 6.4. The concentration of OM was low (ranging from 1 to 2.5%), with a mean of 2.13%. Total N was relatively low (ranging from 0.05 to 0.01%) with a median of 0.09%, though the mean was 0.11%. Available P_2_O_5_ (40.08 kg ha^-1^) and K_2_O (110.61 kg ha^-1^) were within their respective medium ranges. Between the two micronutrients measured, Zn was very low (range: <0.25 mg kg^-1^) with a median of 0.07 mg kg^-1^ and a mean of 0.08 mg kg^-1^, while B was at medium (ranging from 0.5 to 1.2 mg kg^-1^) with a median of 0.99 mg kg^-1^, though the mean was 1.03 mg kg^-1^.

Among the soil chemical properties, OM, N, K_2_O, B, and Zn were found to be not normally distributed due to higher values of skew and kurtosis. Those datasets were then subjected to logarithmic transformation to narrow down the skew and kurtosis values (Table 2) and the transformed datasets were subsequently used in the spatial analysis.

### Digital soil maps using kriging

**Fig 2:**
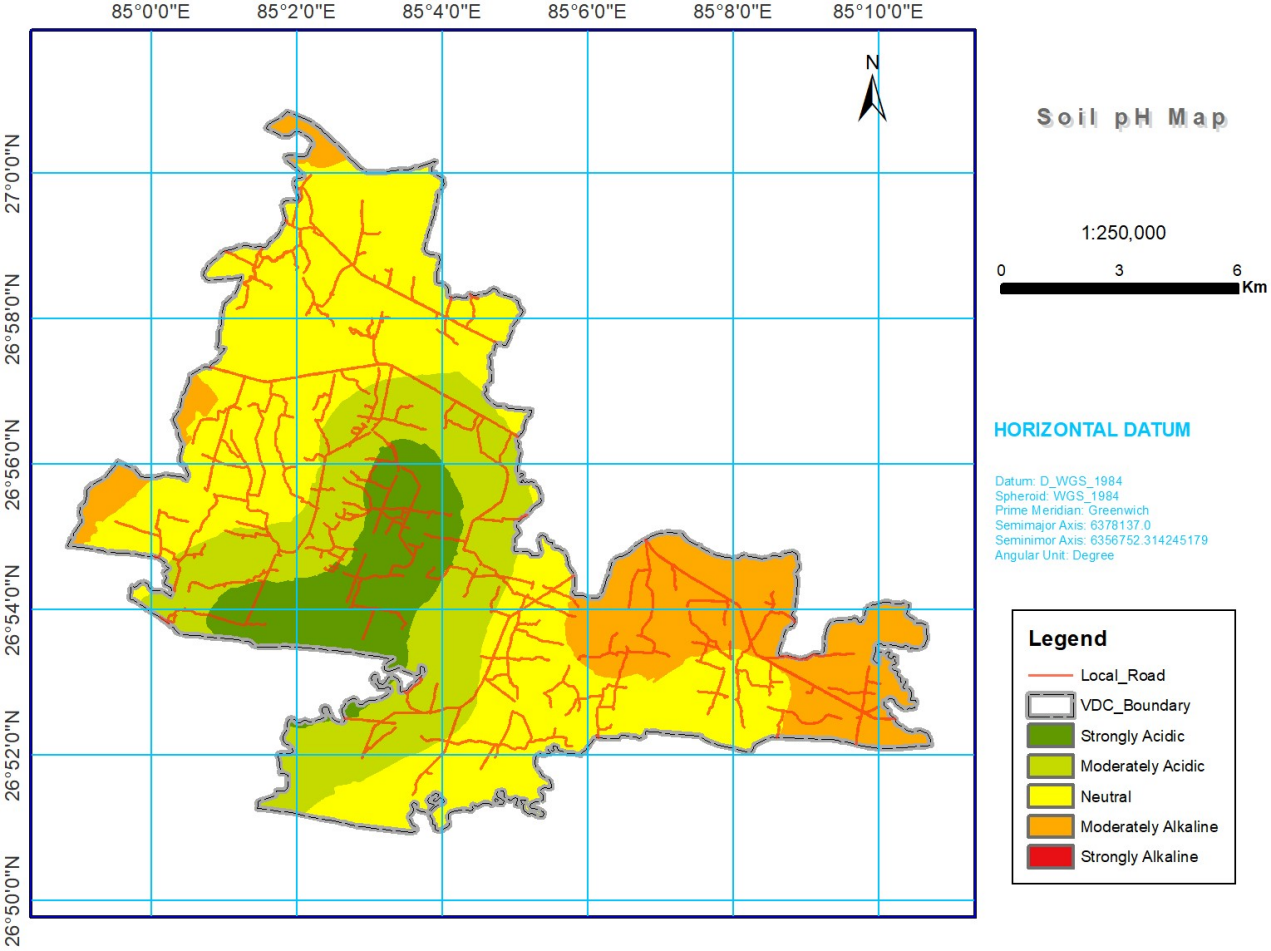
Soil pH spatial variability map in southern part of Bara district, Nepal. Most of the study area was with moderately alkaline (30.69%) followed by moderately acidic (22.91%) and neutral (22.80%) pH. Strongly alkaline was present in about 2.5% of total area but could not see in the variable map.

Digital maps of soil chemical properties were produced by using kriging on the log transformed dataset, and the results (shown in Figs 2 through 8) were grouped into various classes based on the range representing their magnitude in the soil. The estimated area of each class is given in Table 4.

**Fig 3:**
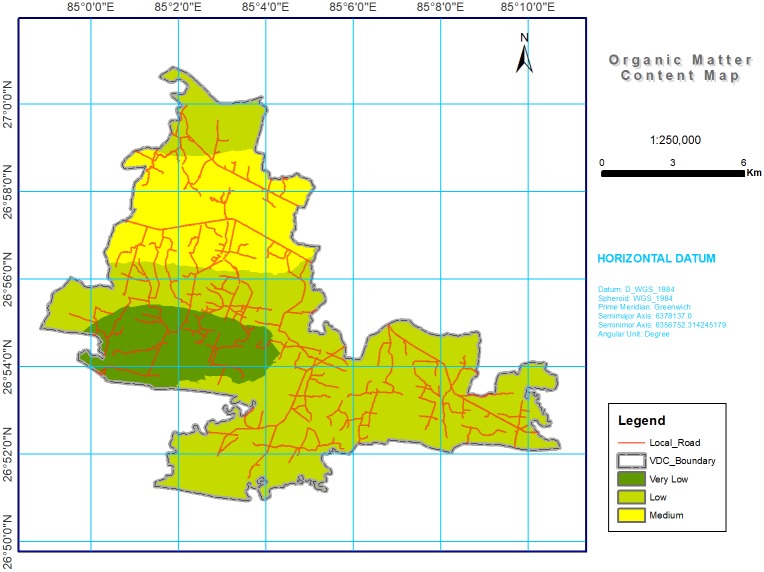
Soil OM spatial variability map in southern part of Bara district, Nepal. Most of study area was with low (48.80%) and very low (26.88%) OM content.

**Fig 4:**
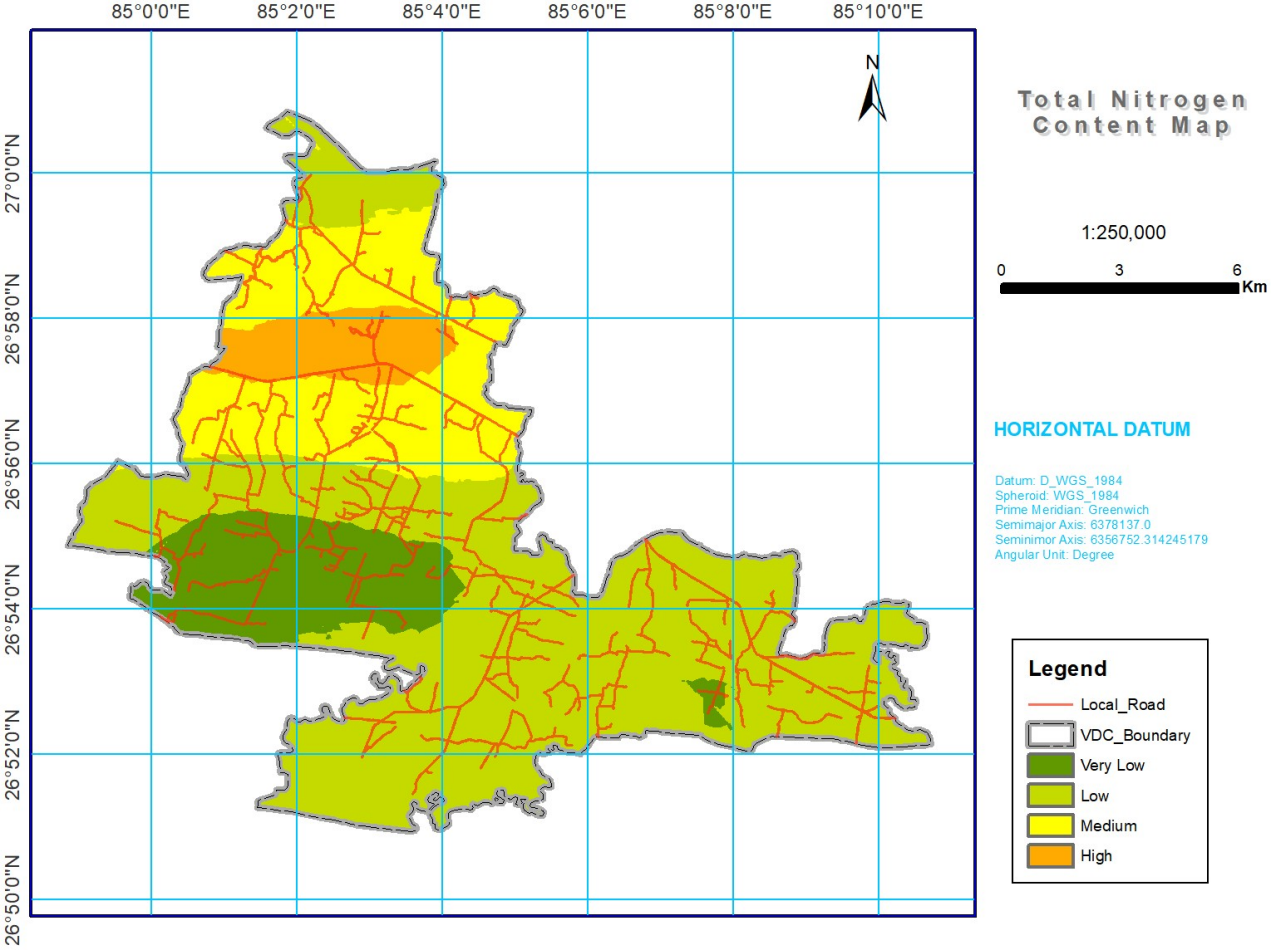
Soil N spatial variability map in southern part of Bara district, Nepal. Most of study area was with low (50.86%) for total N content.

**Fig 5:**
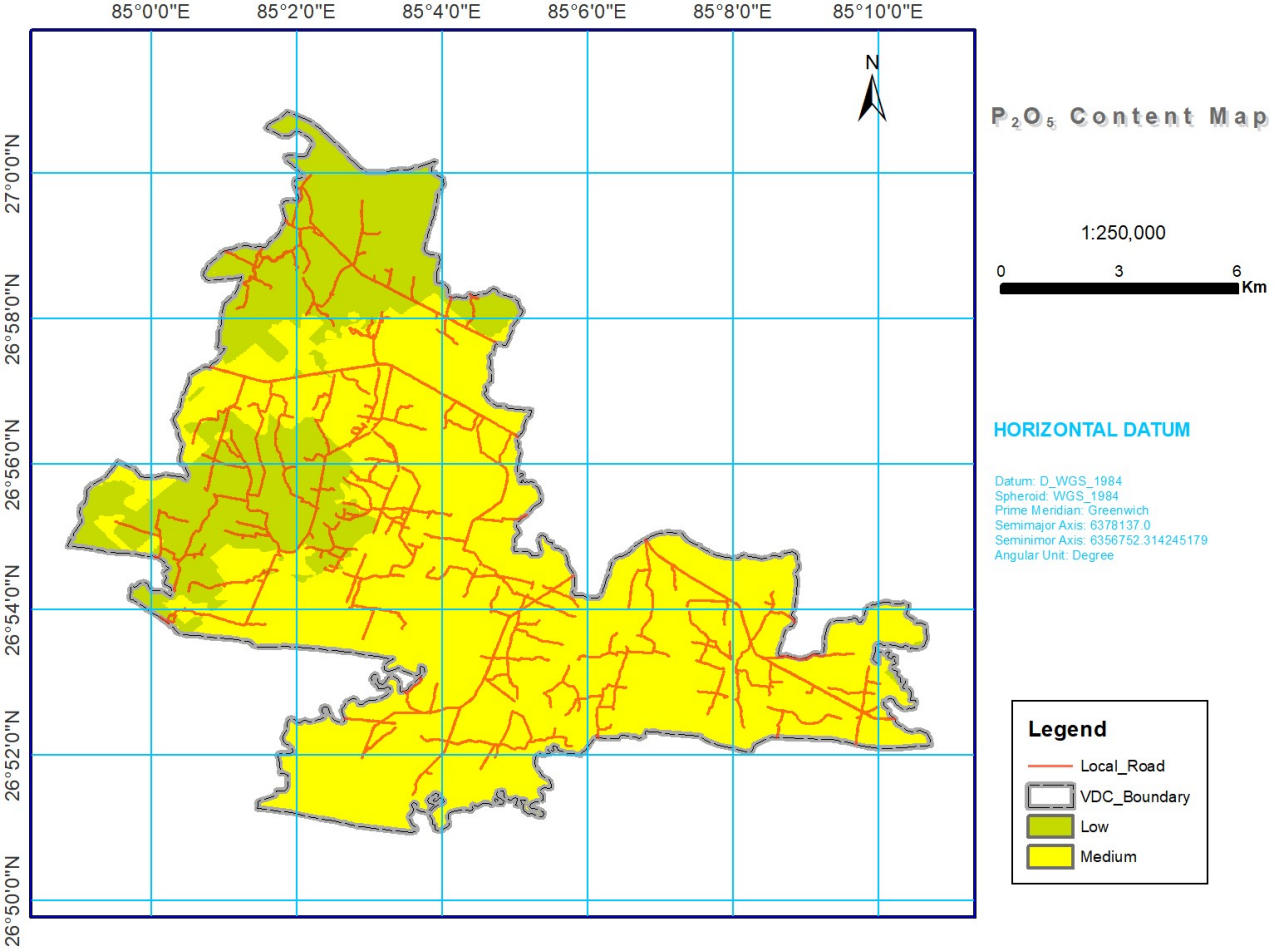
Soil P_2_O_5_ spatial variability map in southern part of Bara district, Nepal. Most of study area was with medium (42.95%) and low (29.88%) for available P_2_O_5_.

**Fig 6:**
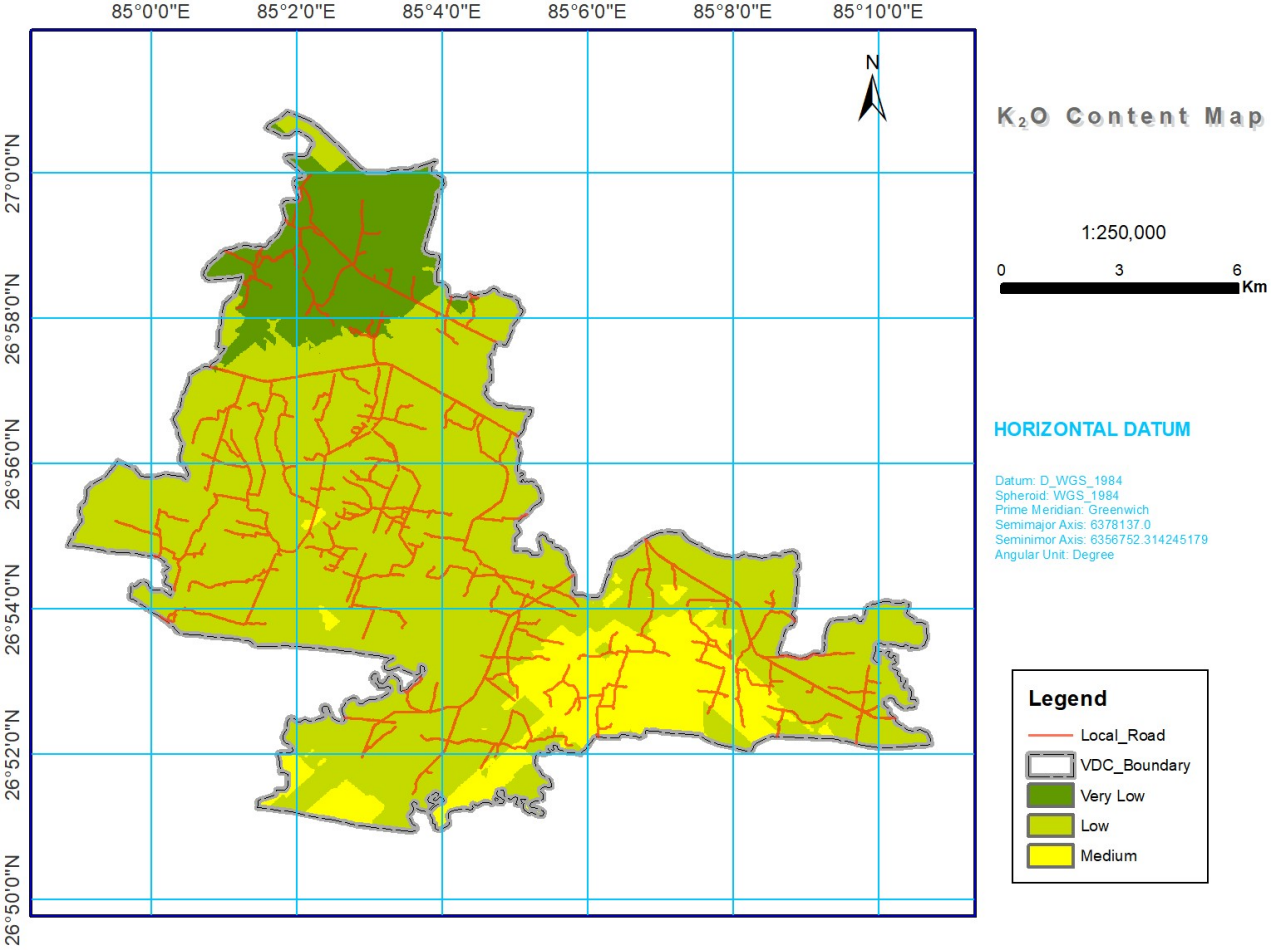
Soil K_2_O spatial variability map in southern part of Bara district, Nepal. Most of study area was with low (41.19%), followed by very low (30.77%) and medium (21.79%) for available K_2_O.

**Fig 7:**
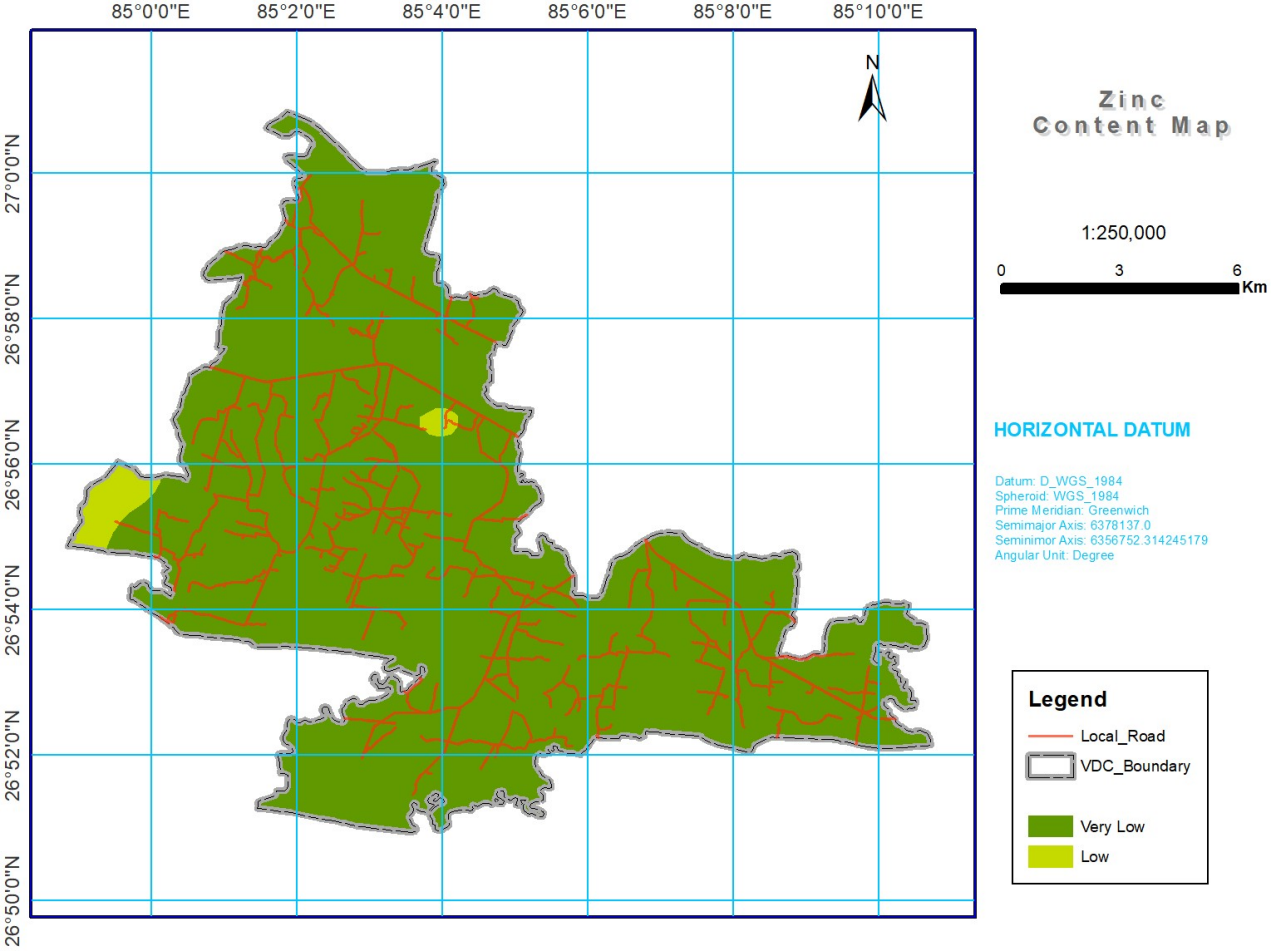
Soil Zn spatial variability map in southern part of Bara district, Nepal. Almost of the study area was with very low (98.33%) for Zn content.

**Fig 8:**
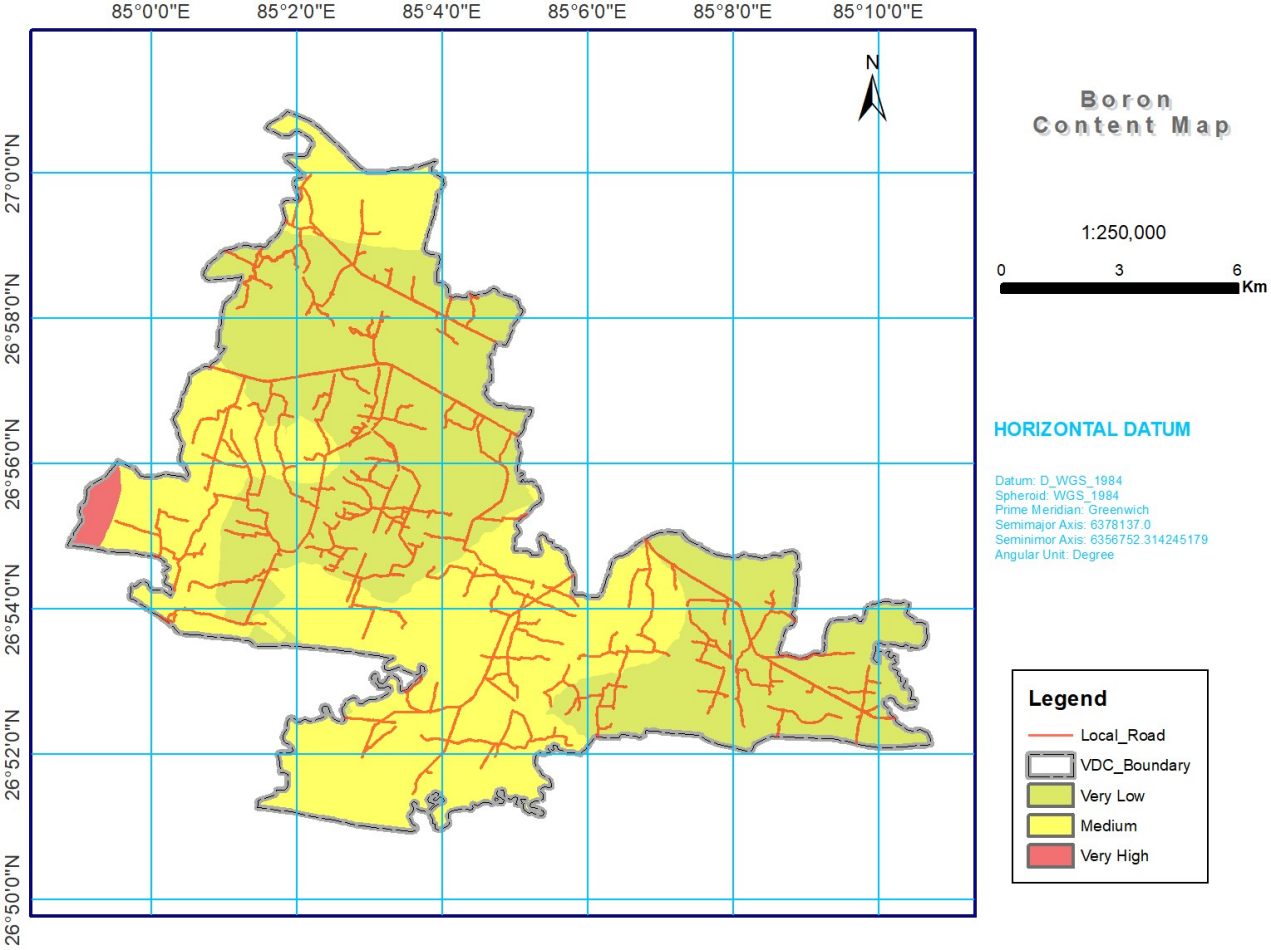
Soil B spatial variability map in southern part of Bara district, Nepal. Most of study area was with low (55.52%) and medium (43.21%) for B content.

**Table 4:** Areas under different soil categories based on soil fertility parameters.

Parameter	Unit	Rating	Class	Area (ha)	% of total area
pH		<5.5	strongly acidic	2643.34	21.12
		5.5–6.2	moderately acidic	2868.06	22.91
		6.2–7.0	neutral	2853.46	22.80
		7.0–7.8	moderately alkaline	2841.68	30.69
		>7.8	strongly alkaline	309.82	2.48
OM	%	<1	very low	3364.75	26.88
		1–2.5	low	6108.35	48.80
		2.5–5.0	medium	1822.53	14.56
		5.0–10.0	high	1220.73	9.75
		>10.0	very high	-	-
N	%	<0.05	very low	2457.94	19.64
		0.05–0.10	low	6365.32	50.86
		0.10–0.20	medium	2050.26	16.38
		0.20–0.40	high	1642.84	13.13
		>0.40	very high	-	-
P_2_O_5_	kg ha^-1^	<10	very low	335.22	2.68
		10–30	low	3739.98	29.88
		30–55	medium	5375.94	42.95
		55–110	high	2772.24	22.15
		>110	very high	292.98	2.34
K_2_O	kg ha^-1^	<55	very low	3851.55	30.77
		55–110	low	5154.92	41.19
		110–280	medium	2726.93	21.19
		280–500	high	411.43	1.67
		>500	very high	371.53	2.60
Zn	mg kg^-1^	<0.25	very low	12307.92	98.33
		0.25–0.5	low	208.44	1.67
		0.5–1.0	medium	-	-
		1.0–2.0	high	-	-
		>2.0	very high	-	-
B	mg kg^-1^	<0.2	very low	-	-
		0.2–0.5	low	6949.26	55.52
		0.5–1.2	medium	5408.10	43.21
		1.2–2.0	high	-	-
		>2.0	very high	159	1.27

#### Soil pH

Soil pH varied from strongly acidic (< 5.5) in 21.12% to strongly alkaline (> 7.8) in 2.48% of the total area (Table 4 and Fig 2). These results are in agreement with those reported in a recent study of soils of the Terai region [22, 45]. The variation in soil pH could be attributed to the nature of the alluvial parent material, micro topography, and the type and history of fertilizer used [46]. The losses of basic cation and other nutrients through erosion and leaching leaves the hydrogen and aluminum ions that can cause soil acidity [47]. Management practices such as crop nutrient uptake and harvest without replenishment [48] and poor crop residue management lowers the pH and leads to low levels of soil OM [28, 49].

Urea (46% N) and di-ammonium phosphate (18% N and 46% P) are the most commonly used fertilizers by Nepalese farmers. The national average for the use of chemical fertilizer has increased dramatically from 16.7 kg ha^−1^ in 2002 to 67.4 kg ha^−1^ in 2014 [50]. Of the major fertilizer nutrients, types of N fertilizer containing ammonium-N are the main factors affecting soil pH. As the ammonium-N in fertilizers undergoes nitrification, hydrogen ions are released, which can increase acidity [51], and Tripathi and Shrestha [52] reported an increase of acidity up to 4.1 (in 2000) from 5.6 (in 1997) after the application of fertilizers at four locations in western Nepal.

Plant growth and most soil processes are favored by a specific pH range. The low pH leads to Al and Mn toxicity, along with deficiency and/or unavailability of plant nutrients such as P, Ca, K, Mg, and Mo as observed by Dembele *et al*. [53] and Tisdale *et al*. [54]. To produce a sustained crop growth and yield, efforts should be made to increase the pH, which can be addressed through liming and OM management or adoption of the acid tolerant crops.

#### Soil organic matter

Soil OM was relatively low (1 to 2.5%) in the majority (48.8%) of the study area, followed by very low (<1%) in 26.88% (Table 4 and Fig 3). The low organic content in the soils can generally be accounted for through the general sparse vegetation and competing use of crop residue as animal feed which then constrains their return to the soil [55–56]. A study conducted in the Dhading district of Nepal in 2003 showed that 37% of crop residue was used to feed livestock, 35% was used as fuel, 15% was burnt on lands, and the remaining 13% was incorporated into the soil through methods other than burning [57].

Another possible reason for low OM is a high soil OM decomposition rate resulting from soil and higher air temperature that decreases soil organic carbon (SOC). The SOC is affected by the addition of farm yard manure (FYM), tillage, and cropping pattern [58–59]. Around 14.56% of the study area revealed medium (2.5 to 5%) levels of soil OM, which could be due to waterlogged conditions, leading to shallow rooting and the confinement of biological activity to the upper soil layer. Similar results were reported by Shrestha et al. [60] for the soils of lowland irrigated rice fields in Nepal.

Among Nepalese farmers, there is an increase in the use of chemical fertilizer in agriculture, though this increase is not being matched by an increase in the use of organic manure (manures, organic fertilizers, compost, or other soil improvers) [61]. The present rate of organic manure application is 2.5 to 3 t ha^-1^ for soil fertility management [62], with an estimated composition of 0.5% N, 0.2% P, and 1.25% K on a dry weight basis, far below the global average and a rate that may not meet crop demand on a long-term basis [63–64]. As OM decreases, it also decreases available N, P, K, and some micronutrients [65]. Zhao *et al*. [66] reported that this low level of OM is indication of soil degradation and a high risk of soil erosion. Farmers should be encouraged to add much crop residues to the soil along with manure and compost.

#### Total nitrogen

Usually, N has a greater effect on crop growth, crop quality, and yield. However, N was deficient in most of the areas with values <0.1 (low and very low) recorded in 70.5% of the total area (Table 4 and Fig 4). The variation in N content in different parts of the study area may be related to soil management, application of FYM and applied fertilizer to previous crops, etc. [67]. The acute deficiency of N is due to low OM content, increased rate of mineralization, and insufficient application of N fertilizer to nutrient exhausting crops like wheat and maize [46]. The rate of soil OM decomposition and N mineralization holds complex interactions with the microbial population and other environmental factors, mainly soil moisture and temperature. A field with 40 kg N ha^-1^ of soil nitrate build-up led to the loss of N from the entire field when the soil, which contained moisture levels > 46%, filled pore space at the onset of the monsoon rains in a lowland field in the west central part of Chitwan, Nepal [68].

#### Available phosphorus

The available P_2_O_5_ was medium in 42.95% and low in 29.88% of the study area (Table 4 and Fig 5). The low level of OM may account for the low level of available P_2_O_5_ in the soils. However, the relatively higher availability of P_2_O_5_ observed in some areas may be due to the dissolution of Ca-P under neutral soil reaction under cultivated conditions [69–70]. Phosphorous is more directly affected by soil pH than other major plant nutrients such as N, K, and S; for example, at alkaline values, greater than pH 7.5, the HPO_4_^2-^ phosphate ions tend to react quickly with calcium (Ca) and magnesium (Mg) to form less soluble compounds. At acidic pH values, the H_2_PO_4_^-^ phosphate ions react with aluminum (Al) and iron (Fe) to again form less soluble compounds [71]. Soils with inherent pH values between 6 and 7.5 are ideal for P availability. Besides pH, the amount of OM and the placement of P fertilizers also control the availability of P_2_O_5_, whereas erosion and runoff are associated with its loss from soil. Studies from many developed countries have shown that the use of flue gas desulfurization gypsum, a source of Ca and S, can be used as a soil amendment, especially to reduce soil and soluble P loss from agricultural fields and improve acidic soils [72]. Hence, whether or not farmers attempt to adjust pH levels, it is important to understand methods to increase the availability and use of added nutrients [73].

#### Available potassium

The available K_2_O was at low levels in the majority of the study area (Table 4 and Fig 6). Soil pH also affects the availability of K_2_O. When soil pH is greater than 7, the greater Ca concentration increases the K availability through the displacement of exchangeable K by Ca. Conversely, when soil pH is less than 5.5, the reduction in Ca concentration reduce the K availability. In addition, low levels of OM due to low clay content, high hydraulic conductivity, and possible nutrient losses through leaching and erosion without replenishment also reduces the K level [74]. Water for irrigation to many of these study areas comes from Nepal’s rivers, which are flooded during monsoon season and carry heavy sediments (for example, mica) a source of exchangeable K [75]. However, due to year-round cropping practices, there is very little time for K to release from sediments and remain in the exchangeable site [76]. This could be another reason why the majority of the study area included low amounts of K_2_O.

#### Zinc and boron

The micronutrient Zn was low and B was at medium level throughout the study area (Table 4 and Figs 7 and 8), possibly due to unfavorable soil pH (moderately alkaline in 30.69% of the total study area), intensive cropping, the use of high yielding varieties, and different fertilizer application strategies practiced by smallholder farmers. The Khaira disease (leaf bronzing) in rice due to Zn deficiency [77–78] and sterility in wheat induced by an inadequate B supply [79–82] are major concerns in the study area. In a study of micronutrient deficiencies in grain legumes, Srivastava et al. [83] found that B severely restricted the growth of lentils (*Lens culinaris* M.), chickpeas (*Cicer arietinum* L.), and pigeonpeas (*Cajanus cajan* L.) in the Terai region. Since rice is the major staple crop in Nepal, farmers use zinc sulphate (ZnSO_4_) before transplanting or sowing at the time of land preparation, along with a combination of ZnSO_4_ and lime during the growing stage, if the crop is infected [84].

#### Farmers’ practice and use of digital soil maps

In many developing countries including Nepal, soil fertility management recommendations are solely based on soil types and agro-ecological zones. Details about soil pH range and the recommended agricultural lime rate, as well as the recommended doses of chemical fertilizers for specific crops in Nepal are given in Pandey *et al*. [22]. Despite the advisory recommendation made from research, farmers do not apply balanced doses of fertilizer, and instead use mostly acid forming nitrogenous fertilizers.

Most of the farmers apply FYM to their lands at the same rate as it is produced. The practice for FYM preparation and application is not an improved one because farmers dump FYM in open spaces and expose it to the sun, wind, and rain for several days before ploughing [85]. Farmers also follow nutrient-exhaustive high-yielding crop varieties under intensive cropping all year-round, leading a heavy loss of nutrients after every harvest. Therefore, a balanced rate of chemical fertilizers and organic manures must be applied every year.

Maps that characterize the spatial distribution of each soil property can be produced using kriging to group individual fields into potentially low- and high-productivity areas. Hence, management strategies to enhance soil nutrients could be implemented in the study area by using these DSMs as a guide [86], such as famers following fertilizer recommendations based on buildup and maintenance levels. Normally, nutrient values that are at low levels require relatively higher amount of fertilizer application; therefore, these DSMs may lead to proper understanding of existing farm soils by allowing easier management and maintaining the sustainability of productivity. This research sets a precedent for future DSM in other parts of the country.

### Geostatistics for soil chemical properties

#### Semivariogram analysis

The semivariogram model and some of the geostatistical parameters of soil chemical properties are shown in Table 5. Based on the lowest root mean square error (RMSE), different theoretical semivariogram models were selected for the significant fit of soil chemical properties [87]. An exponential model provided the best fit to the semivariogram of pH, OM, N, and Zn. The spherical model was the best fit to the semivariogram of K_2_O and B, whereas Gaussian was the best fit for P_2_O_5_. Many findings suggest that the exponential model is the most suitable for assessing spatial variability in soil chemical properties [88–92] because it explains the maximum variability in the spatial dataset [93–94].

**Table 5:** Semivariance analysis of spatial structure in soil chemical properties.

Property	ME	RMSE	Model	Range	Lag size	Nugget	Partial	Sill	Nugget/Sill	Sp. D
pH	-0.03	0.057	E	5132	635.35	0.57	0.43	0.99	0.57	M
OM	0.01	0.026	E	4951	682.59	0.16	0.39	0.57	0.31	M
N	0	0.012	E	5209	675.64	0.16	0.37	0.53	0.3	M
P_2_O_5_	0.08	0.109	G	5038	661.78	0.15	0.14	0.29	0.513	M
K_2_O	0.01	0.061	S	5831	661.93	0.45	0.13	0.58	0.78	W
Zn	0	0.06	E	5945	800.11	0.33	0.03	0.35	0.92	W
B	-0.03	0.43	S	5113	634.73	0.04	0.013	0.06	0.77	W

ME = mean error, RMSE = root mean square error, E = Exponential, G = Gaussian, S = Spherical, M = Moderate, and W = Weak. Unit for range and lag size, m.

Table 6 shows that Sp. D of soil parameters ranged from 0.3 (in N) to 0.92 (in Zn). There was a moderate (in N, OM, P_2_O_5_, and pH) and weak (in K_2_O, Zn, and B) Sp. D. of the kriging model, which could be attributed to external factors such as variable rates of fertilizer application and incorporation of amendments by farmers within a cropped region. The ranges of spatial dependencies were large and vary between 4951 m for OM to 5945 m for P_2_O_5_ indicating that the optimum sampling interval varies greatly among different soil properties. Determination of the range values provides an idea of the correlation between different sampling locations, along with the maximum spatial dependence distance between them [95]. Fluctuation in the range with different lag sizes indicates that spatial structure may merely be regarded with a single model for semivariogram [96]. This difference may not be important for semivariance calculation, but it may be important if the purpose is to understand the underlying spatial structure of the data [97].

**Table 6:** Test of significance of pattern analysis for selected soil chemical properties.

	pH	OM	N	P_2_O_5_	K_2_O	Zn	B
Moran’s Index	0.675	0.625	0.633	0.152	-0.073	0.333	0.064
Variance	0.013	0.013	0.013	0.013	0.012	0.012	0.004
*z*-score	6.043	5.638	5.714	1.43	-0.599	3.148	1.118
*p*-value	0.000	0.001	0.003	0.153	0.549	0.002	0.264

#### Spatial autocorrelation

The analysis of spatial autocorrelation based on Global Moran’s I Index was used to identify the spatial pattern soil chemical properties that may be dispersed, random, or clustered based on feature locations and attribute values simultaneously [42], as presented in Table 6. The hypothesis for the pattern analysis was that the soil chemical properties (including pH, OM and some nutrients) across the study area were randomly distributed.

According to ESRI [98], for the theory of random patterns, when the *p*-value is very small (in this study, *p* < 0.05) and the *z*-score is either very high or very low (1.96 < *z* and *z* < −1.96), the spatial pattern is not likely to reflect a random form of distribution. A positive Moran’s I index value indicates the neighboring values are similar, suggesting spatial dependency. A negative Moran’s I index value indicates the neighboring values are dissimilar, suggesting inverse spatial dependence. A Moran’s I index value of zero implies a lack of spatial pattern [99–101]. With the exception of K_2_O, all other soil variables had a positive Moran’s I index for their spatial pattern (Table 6).

Test of significance for values returned by the analysis of the major soil chemical properties indicated that pH, OM, N and Zn showed clustered distributions in the study area, with low levels clustered at one location and high levels at the other (no figures included). On the other hand, the pattern of distribution of P_2_O_5_, K_2_O and B did not appear significantly different from a random distribution at *p* < 0.05.

## Conclusions

The application of the geostatistical approach, including descriptive statistics and semivariogram analysis, improved the description of spatial variability for soil chemical properties at 0 to 15 cm depth on a field scale. The descriptive statistics showed that most of the measured soil chemical variables were skewed and non-normally distributed and the available K_2_O data were highly variable (5 to 696 kg ha^-1^). Geostatistical interpolation identified that exponential, spherical, or Gaussian models provided the best fit to the semivariograms, depending on the soil chemical variable and, in general, showed weak or moderate spatial dependency for all of the variables. The kriging maps of soil chemical properties were found effective in explaining the distribution of soil properties in non-sampled locations based on sampled data. These maps aid farmers in to making efficient management decisions based on their proper understanding of the conditions of existing farm soils. These results show geostatistical analysis using kriging is an effective prediction tool for exploring the spatial variability of soil nutrients, and we recommend this tool for future soil sampling campaigns in Nepal.

## Supporting information

S1 File An excel file include coordinates of 109 sampling locations and report of different soil chemical properties. (CSV)
